# Antibiotic Use for Febrile Illness among Under-5 Children in Bangladesh: A Nationally Representative Sample Survey

**DOI:** 10.3390/antibiotics10101153

**Published:** 2021-09-24

**Authors:** Nora Samir, Md. Zakiul Hassan, Md. Abdullah Al Jubayer Biswas, Fahmida Chowdhury, Zubair Akhtar, Raghu Lingam, Sayera Banu, Nusrat Homaira

**Affiliations:** 1 Discipline of Paediatrics, School of Women’s and Children’s Health, Faculty of Medicine, The University of New South Wales, Sydney 2031, Australia; n.samir@unsw.edu.au (N.S.); r.lingam@unsw.edu.au (R.L.); n.homaira@unsw.edu.au (N.H.); 2 Programme for Emerging Infections, Infectious Disease Division, International Centre for Diarrhoeal Disease Research, Bangladesh (icddr,b), Dhaka 1212, Bangladesh; jubayer.biswas@icddrb.org (M.A.A.J.B.); fahmida_chow@icddrb.org (F.C.); zakhtar@icddrb.org (Z.A.); sbanu@icddrb.org (S.B.); 3 Nuffield Department of Medicine, University of Oxford, Oxford OX3 9DU, UK; 4 Respiratory Department, Sydney Children’s Hospital, Sydney 2031, Australia

**Keywords:** febrile illness, under-5 aged children, antimicrobial use, antibiotic, Bangladesh

## Abstract

Fever in children under five years of age is a common and predominantly self-limiting sign of illness. However, in low- and middle-income countries, antibiotics are frequently used in febrile children, although these children may not benefit from antibiotics. In this study, we explored the prevalence of, and factors associated with, antibiotic use in children under five years old with febrile illness in Bangladesh. We analysed data from the 2017–2018 Bangladesh Demographic and Health Survey to determine the prevalence of antibiotic use in children under five years of age with a febrile illness. We used a causal graph and performed a multivariable logistical regression to identify the factors associated with antibiotic use in children under five years old with febrile illness in Bangladesh. Of the 2784 children aged less than five years with fever included in our analysis, 478 (17%, 95% CI 15% to 19%) received antibiotics. Unqualified sources, including unqualified providers and pharmacies, contributed to 60% of antibiotic prescriptions in children with fever, followed by the private medical sector (29%) and the public sector (23%). The highest use of antibiotics was found in children under six months of age (25%). Children with parents who completed secondary or higher education were more likely to receive antibiotics (adjusted OR (aOR): 2.61 (95% CI 1.63 to 4.16)) than children whose parents did not complete primary education. Educational interventions promoting rational use of antibiotics and improved regulations governing over the counter purchase of antibiotics in Bangladesh may improve antibiotic dispensing practices.

## 1. Introduction

Febrile illnesses in children under five years of age are common. On average, children worldwide experience three to six episodes of febrile diseases per year [1,2]. This rate is even higher in children living in low and middle-income countries (LMICs) [3,4]. Fever is also the single most common reason for children to be seen by medical practitioners and is one of the most frequent presenting symptoms in emergency department visits [2]. Studies suggest most acute febrile illnesses in children requiring ambulatory care visits have a viral aetiology and do not require antibiotics [5]. These illnesses include acute respiratory infections (ARIs), accounting for 50–75% of febrile presentations at outpatient clinics, and gastroenteritis, accounting for 10–25% of febrile illnesses. Clinical practice guidelines (CPGs), such as the Integrated Management of Childhood Illness (IMCI), aim to standardise the symptomatic management of fever in children [6]. These guidelines promote the rational use of antimicrobials by recommending antibiotics be exclusively prescribed to children who are presumed to have a disease that can be treated using antibiotics, based on World Health Organisation (WHO) recommendations [7]. Despite these recommendations, healthcare providers erring on the side of caution continue to prescribe antibiotics frequently to patients who may not have an illness with bacterial aetiology [7]. Factors contributing to the irrational use of antibiotics in febrile children include diagnostic uncertainty, inadequate knowledge and experience, and pressure from anxious parents [8,9,10].

Bangladesh is a LMIC where more than half of the antibiotics used in children are thought to be inappropriately prescribed, dispensed, or sold [11,12,13]. This inappropriate antibiotic usage can be attributed to several factors within Bangladesh’s pluralistic healthcare system, which disparate actors govern: government, private sectors, non-governmental organisations (NGOs), and informal sectors, including unqualified providers and pharmacies [14]. One of the significant contributors to the inappropriate use of antibiotics in Bangladesh is unregulated ‘drug shops’ and unqualified providers who supply antibiotics to a large proportion of the population, often in incorrect doses [15]. This overuse of antibiotics risks several consequences at the individual, system, and population levels. Among these potential consequences is the emergence of antimicrobial resistance (AMR), increased risk of developing chronic diseases such as asthma, increased healthcare costs, and wasted resources [8,16]. In line with the WHO Global Action Plan on Antimicrobial Resistance (GAP), Bangladesh has taken the first step to address AMR [16]. In 2018, in collaboration with other development partners, the Government of Bangladesh undertook a situational analysis. It published the National Action Plan (NAP) on AMR containment [17]. The objectives of the NAP include promoting the rational use of antibiotics, establishing a multi-sectoral approach for the planning and implementation of antimicrobial resistance containment activities, and strengthening regulatory provisions and surveillance systems for AMR containment. To achieve these objectives and monitor trends over time, population-level baseline data on the use of antibiotics for febrile illnesses—one of the most common presenting symptoms in children—is crucial.

Several studies have examined antibiotic dispensing patterns in Bangladeshi children hospitalised for diarrhoeal disease and pneumonia, but there are limited data on the overall use of antibiotics for febrile illnesses [12,18,19,20,21,22,23,24]. Additionally, hospital-based studies provide data on the use of antibiotics in small groups of children with more severe than average symptoms of diseases that may warrant antibiotic use. Still, such studies do not provide information that accurately reflects the general population. There are also limited population-level data on the socio-demographic factors associated with antibiotic usage for febrile illness in children, and such data may inform targeted interventions that improve antimicrobial stewardship. Therefore, we examined the prevalence and associated factors with antibiotic usage in children under five years old with febrile illness in Bangladesh utilising nationally representative, population-based sample survey data.

## 2. Results

### 2.1. Participant Socio-Demographic Characteristics

Of 8421 children surveyed, 2784 (33%) had experienced fever in the preceding two weeks. Of 2784 children with fever, 254 (9%) also had symptoms of acute respiratory infection (ARI), and 396 (14%) also had diarrhoea. A total of 478 (17%) children with fever received antibiotics for their illness and were thus included in this analysis. Twenty five percent of children who received antibiotics for their febrile disease were aged less than six months old, and nine percent were between 48 and 59 months. Children of parents with secondary complete or higher level of education had the highest percentage of antibiotic use for febrile illness (Table 1).

### 2.2. Prevalence of Antibiotic Use in Febrile Children

The total percentage of febrile children who were given antibiotics was almost 17%. In addition, statistically significant associations were found between the prevalence of antibiotic usage in febrile children and children’s age, parents’ highest level of education, and first source of treatment (Table 1).

### 2.3. Factors Related to the Use of Antibiotics in Children Who Had a Fever

The results from logistic regression analysis indicate the unadjusted and adjusted odd ratios (ORs) and the 95% confidence intervals (CIs) for the variables associated with antibiotic usage in children under five years old. Children who reported the private medical sector as the first source of antibiotics were 1.66 times more likely (95% CI 1.09 to 2.50) to use antibiotics than children who reported pharmacy as a source of antibiotics (Table 2). The odds of using antibiotic was almost three times (95% CI 1.83 to 4.47) higher in children aged less than six months compared with older children (Table S1). Moreover, children with parents who completed a secondary or higher level of education were 2.61 times (95% CI 1.63 to 4.16) more likely to report the use of antibiotics for children with fever than parents with lower levels of education (Table S2).

## 3. Discussion

Our results showed that around 17% (95% CI 15–19) of children under five years of age with febrile illness received antibiotics. If we extrapolate our estimates to about 16.7 million children in Bangladesh, we estimated that 5.5 million children had the fever, with an estimated 943,186 receiving antibiotics [25]. Our findings are comparable to the proportion of children who received antibiotics for fever in other LMICs, as reported in a recent systematic analysis of 132 national surveys from 73 LMICs (49.6%; range 12.2–82.6%) [26].

Nearly 60% of antibiotics were obtained from unqualified sources, including pharmacies and unqualified providers. This is alarming since most pharmacies in Bangladesh are run by unqualified practitioners who provide antibiotics without a prescription [27]. Several studies have reported the overuse of antibiotics obtained from unqualified providers and drug shops in Bangladesh, and 80–90% of drug shops and medicine vendors operate without a license [8,13,19,28]. Additionally, the adequate supervision and inspection of drug outlets are complicated by the limited availability of drug inspectors (i.e., one drug inspector supervises 2236 pharmacy shops) [6]. Initiatives, such as the ‘model pharmacy’, which accredit pharmacies for meeting high standards of practice, may help to reduce over the counter purchases of antibiotics [29].

Our data indicated that 29% of antibiotic use for febrile illness came from the private medical sector and 23% from the public sector. Additionally, children who went to the private sector were significantly 1.66 times more likely (95% CI 1.09–2.50) to receive antibiotics compared to those who went to a pharmacy for treatment. The unadjusted and adjusted effect size was nearly similar, suggesting that any child’s age in months, sex, type of place of residence, wealth index, parents’ highest level of education had little impact on the association. These results are consistent findings from previous literature, which indicates that in LMICs, private healthcare providers, particularly informal providers, contribute to a significant percentage of antibiotic dispensing [30]. Consistent with our findings, several studies conducted in LMICs have revealed higher paediatric antibiotics prescribed in the private sector than in the public sector [31]. This trend could be explained by public health facilities not being able to meet the high demand for their services. Long waiting times and lack of drug availability in Bangladeshi public health facilities may also motivate patients to seek healthcare advice and obtain antibiotics from the private medical sector [8,32].

Our study showed that children whose parents completed secondary and higher education were almost twice as likely to receive antibiotics for fever than children with parents who did not complete primary education. This is contrary to the existing literature on LMICs, which suggests that low parental educational levels are associated with higher use of antibiotics in children [33]. Parents with higher education are often more concerned about their children’s health but do not necessarily have higher awareness of the effects of antibiotic use [34]. This is consistent with our data, which indicates that 46% (132/285) and 27% (78/285) of parents who received antibiotics for children with febrile illness from unqualified sources (such as pharmacies and unqualified providers) had an incomplete secondary education and a completed secondary or higher education, respectively. This is significantly higher than parents with an incomplete primary education who received antibiotics for children with febrile illness from unqualified sources (10% (28/285)). This could explain our finding that antibiotic use was higher in children with more educated parents. Although we cannot comment on whether parents’ education is a predictor of inappropriate use of antibiotics, it is still crucial to improve public awareness on appropriate antibiotic use through community-based interventions [30].

Our analysis used a population-based, national representative dataset of more than 8400 families and used standardised data collection methods. Thus, the findings can be extrapolated at a national level. Despite this study’s strengths, it has certain limitations. The most important limitation of our study is the lack of available information on fever aetiology. As such, we could not assess appropriate versus inappropriate antibiotic use. However, we aimed to determine the prevalence of use of antibiotics. Further, our outcome measure was based on self-reported data, which may have led to recall bias, such that parents did not accurately report types of symptoms and treatments. This potential influence of recall bias was likely reduced by trained BDHS interview teams asking parents to show the packets of medicines dispensed to their children.

## 4. Materials and Methods

### 4.1. Data Sources

We used data from the most recent 2017–2018 (published in 2021) Bangladesh Demographic and Health Survey (BDHS), a nationally representative, cross-sectional household survey that collects child health data, including treatment practices and contact with health services for childhood illnesses [35]. These data are anonymised and publicly available for secondary analysis on request. 

### 4.2. Study Population and Sampling Strategies

The BDHS used a two-stage stratified sampling design, whereby 675 enumeration areas (EAs) were sampled in the first stage of sampling using a probability proportional to size approach. EAs are geographical areas that include 113 households from a town, a small village, or a section of a larger village. The Bangladesh Bureau of Statistics compiled a list of EAs for the People’s Republic of Bangladesh’s 2011 population census. BDHS 2017–2018 utilised that list of EAs as the sampling frame—250 EAs were selected from urban areas and 425 from rural areas. The second stage of sampling included selecting a systematic sample of 30 households per EA to provide estimates for key factors that were more statistically trustworthy for the entire population. Finally, the survey was administered to a total of 20,160 households and completed in 19,584 residential households. In these households, 20,376 women aged 15–49 were identified, and 20,127 were interviewed by trained personnel. The response rate was approximately 99%. To obtain information on antibiotic usage, mothers were asked about the history of any febrile illness and treatment sought for that febrile illness in children aged 0–59 months in the two weeks preceding the interview. We included all children who had a history of taking antibiotics in the preceding two weeks due to fever from any cause and could have included but not limited to fever due to ARI or diarrhoea. The Bangladesh Demographic and Health Survey 2017–2018 report contains detailed information on the sampling strategies and execution [35].

### 4.3. Data Collection Tools

Household Questionnaire, Women’s Questionnaire, Biomarker Questionnaire, Verbal Autopsy Questionnaires, Community Questionnaire, and Fieldworker Questionnaire were among the six types of questionnaires used in the 2017–2018 BDHS. The Women’s Questionnaire was used to gather data on background demographic features, fertility, family planning, breastfeeding, prenatal and postnatal treatment, infant immunisation, child health and nutrition, and husband’s characteristics. Each of the six types of questionnaires was adapted, pretested, and validated in the context of Bangladesh. The detailed questionnaires are publicly available [35].

### 4.4. Data Collection

A trained interviewing team collected data for the BDHS between 24 October 2017 and 15 March 2018. Each team was led by a male supervisor, assisted by a female field editor, and comprised five female interviewers, two health technicians, and one logistical staff member. The team obtained informed consent from all participants before administrating the interview. The National Institute of Population Research and Training (NIPORT), Ministry of Health and Family Welfare (MOHFW), ICF, and Mitra and Associates quality control team monitored data collection. Data processing was conducted at Mitra and Associates’ offices using Census and Survey Processing System (CSPro) software [35].

### 4.5. Outcome Variable

The outcome variable for our study was the prevalence of antibiotic use for any reported/documented fever in children under five years of age. We used the Women’s Questionnaire to extract data relevant to this outcome. The Women’s Questionnaire asked mothers if their children under the age of five had a fever within the two weeks before the survey’s completion. Participants were then asked where they sought help or care and what kind of fever medication they used. We aggregated data on all the antibiotics for which information was collected (i.e., Beta-lactum, Macrolides, Quinolone, Cephalosporin, Cotrimoxazole, Gentamycin, and Metronidazole) to calculate the prevalence of antibiotic use [35].

### 4.6. Explanatory Variables

We investigated seven associated factors with antibiotic usage in children with febrile illnesses based on existing literature [13,26,31,32,36,37,38,39,40,41,42,43,44]. Explanatory variables utilised in this study are defined in Table 3.

### 4.7. Statistical Analysis

**Figure 1:**
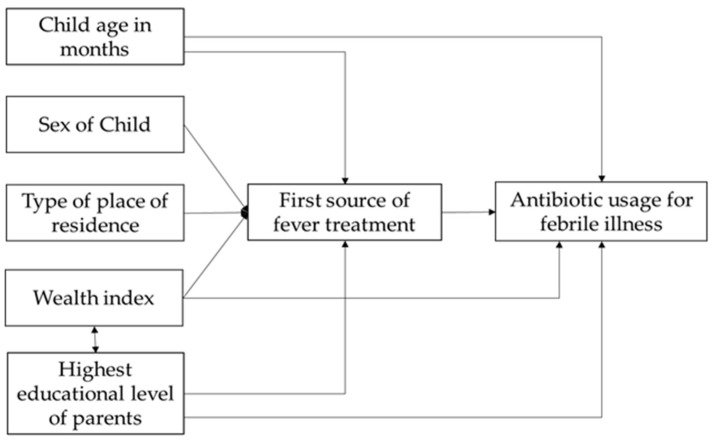
Conceptual framework on associated factors that influenced the children’s antibiotic use for any febrile illness.

We summarised the study participants’ characteristics using descriptive statistics such as frequency, percentage, and cross-tabulation. We performed bivariable logistic regression analysis to determine the crude association between getting antibiotics for febrile illness and selected explanatory factors in children under the age of five. The outputs of bivariable analysis were reported as unadjusted odds ratio (UORs) with 95% confidence interval (95% CI). A conceptual framework technique was used to explore the causal pathways between the outcome variable and the explanatory variables based on empirical knowledge from the findings of previous studies and the authors’ experience (Figure 1) [26,31,32,36,37,38,39,40,42,43,44,45,46,47]. Details about causal graphs are described in Jewell 2003 [48]. The conceptual framework’s arrows indicated the explanatory variable’s causal effect. The first source of treatments influenced children’s antibiotic usage according to their age, sex, parents’ highest level of education, wealth index, and type of residence [34,37,40,42]. We calculated the impact of the first source of treatments using model 1. Likewise, the arrow from children’s age to antibiotic usage depicted the influence of age on their antibiotic usage [34]. We assessed the overall effect of age in model 2. Similarly, the household wealth index and parents’ education affect children’s antibiotic usage and have an interaction effect [41,42,43,44]. We used model 3 to account for the effect of the household wealth index and the parents’ education level. For each multivariable logistic regression model, outputs were estimated and reported as adjusted odds ratios (AOR) with 95% CI. All tests were two-tailed and, at a significance level of 5%, were deemed significant. Stata software package (version 15; StataCorp, College Station, TX, USA) was used to conduct all data analysis.

## 5. Conclusions

In conclusion, the findings of this study provide a comprehensive baseline assessment of antibiotic use for febrile illness among children aged less than five years in Bangladesh across rural and urban locations and among different health care settings. The data from this study may help prioritise, target, implement, and evaluate interventions that improve antibiotic dispensing practices for febrile children in Bangladesh.

## Figures and Tables

**Table 1:** Prevalence of antibiotic usage in febrile children according to both children and parents’ background and treatment-seeking characteristics, 2017–2018 Bangladesh (*n* = 8421).

	Fever in Children Less than Five Years of Age				
	Fever in Children Under the Age of Five (*n* = 2784)	Antibiotic Usage			
		*n*	%	95% CI	*p*-Value
Total	2784	478	17	(15, 19)	
Age in months					
<6	270	66	25	(19–31)	<0.001 *
6–11	372	84	23	(18–28)	
12–23	680	142	21	(17–25)	
24–35	552	80	14	(11–18)	
36–47	472	60	13	(10–17)	
48–59	438	44	10	(7–14)	
Sex of child					
Male	1502	262	17	(15–20)	0.693
Female	1282	216	17	(14–19)	
Type of place of residence					
Rural	714	111	18	(16, 20)	0.241
Urban	2070	367	16	(13–19)	
Wealth index					
Poorest	612	107	17	(14–21)	
Poorer	569	96	17	(14–21)	
Middle	548	96	17	(14–21)	0.495
Richer	594	113	19	(16,23)	
Richest	461	66	14	(11–18)	
Highest education level of parent					
No education	103	12	12	(7–21)	
Primary incomplete	357	37	10	(7–14)	
Primary complete	289	54	19	(14–24)	0.006
Secondary incomplete	1271	221	17	(15–20)	
Secondary complete or higher	759	153	20	(17–24)	
First source of fever treatment					
Public sector	255	58	23	(17–29)	<0.001 *
Private medical sector	465	133	29	(24–34)	
Pharmacy	1277	226	18	(15–20)	
Unqualified provider	294	59	20	(15–26)	
NGO sector	7	1	14	(3–48)	
Other source	90	1	2	(0–11)	
No treatment	398	0	0	0	

* A *p*-value of less than 0.05 was significant.

**Table 2:** Multivariable logistic regression model to determine the association between 1st source of treatment and antibiotic usage in children under five years old for any febrile illness, 2017–2018 Bangladesh.

Model 1—First Source of Treatment Adjusting for Age, Sex, Type of Place of Residence, Wealth Index, Parents’ Highest Level of Education				
	Antibiotic Usage in Children under the Age of Five Year with Fever			
	UOR (95% CI)	*p*-Value	AOR (95% CI)	*p*-Value
Age in months				
<6	2.86 (1.83–4.47)	<0.001 *	3.08 (1.95–4.88)	<0.001 *
6–11	2.58 (1.62–4.11)	<0.001 *	2.25 (1.4–3.63)	0.001 *
12–23	2.33 (1.49–3.65)	<0.001 *	2.31 (1.46–3.66)	<0.001 *
24–35	1.49 (0.91–2.43)	0.110	1.37 (0.83–2.25)	0.215
36–47	1.29 (0.79–2.11)	0.307	1.29 (0.79–2.11)	0.316
48–59	Reference		Reference	
Sex of child				
Female	0.96 (0.77–1.19)	0.693	1.04 (0.82–1.31)	0.752
Male	Reference		Reference	
Type of place of residence				
Rural	1.17 (0.90–1.53)	0.241	1.15 (0.85–1.56)	0.365
Urban	Reference		Reference	
Wealth index				
Poorest	0.90 (0.64–1.27)	0.552	1.19 (0.82–1.75)	0.362
Poorer	0.87 (0.63–1.18)	0.374	1.09 (0.78–1.53)	0.611
Middle	0.90 (0.66–1.24)	0.518	1.02 (0.73–1.43)	0.914
Richest	0.72 (0.51–1.01 )	0.058	0.63 (0.42–0.93)	0.021
Richer	Reference		Reference	
Highest educational level of parent				
No education	1.17 (0.56–2.44)	0.684	1.20 (0.55–2.61)	0.647
Primary complete	1.98 (1.22–3.22)	0.006 *	1.65 (0.98–2.76)	0.058
Secondary incomplete	1.8 (1.19–2.72)	0.006 *	1.65 (1.04–2.62)	0.032 *
Secondary complete or higher	2.15 (1.41–3.28)	<0.001 *	2.13 (1.29–3.49)	0.003 *
Primary incomplete	Reference		Reference	
First source of fever treatment				
Public sector	1.36 (0.94–1.97)	0.096	1.19 (0.73–1.94)	0.485
Private medical sector	1.86 (1.38–2.51)	<0.001 *	1.66 (1.09–2.50)	0.017 *
Unqualified provider	1.17 (0.83–1.66)	0.363	0.92 (0.65–1.30)	0.632
Pharmacy	Reference		Reference	

* A *p*-value less than 0.05 was significant.

**Table 3:** Measurement of explanatory variables.

Variable	Definition
Age in months	The age of the children was computed using the date of birth provided or from the children’s birth history. There were four age groups for children that were less than six months, 6–10 months, 12–23 months, 24–35 months, 36–47 months, and 48–59 months.
Sex of child	Children’s sexes are classified as male or female.
Type of place of residence	The term “place of residence” referred to the region where children resided and was classified as either urban or rural.
Wealth index	A composite indicator summed up the standard of living a household has acquired up to a point. The wealth index was produced by obtaining data on the number of assets owned by each household. A composite factor score was computed through principal component analysis and then divided into five quintiles (poorest: q1; poorer: q2; middle: q3; richer: q4; richest: q5).
Highest educational level of parent	It was a composite variable that indicated the highest education level of father and mother. The variable was categorised as follows: no education (both were illiterate), primary incomplete (either father or mother had education up to grade 5), primary complete (either father or mother completed grade 5), secondary incomplete (any of them had education up to grade 10), secondary incomplete or higher (any of them completed grade 10 or above).
First source of fever treatment	Type of health facility where mothers first sought advice and/or received antibiotics for their children’s fever. This variable included the following categories of health facilities.
	Public sector: This category includes medical college hospitals, specialised government hospitals, district hospitals (DH), mother and child welfare centre (MCWC), upazila health complexes (UHC), union health and family welfare centres (UH&FWC), community clinics (CC), satellite clinics/epi outreach, and other public sector facilities.
	Private medical sector: Private medical facilities such as private medical college hospitals, private hospitals, private clinics, and certified doctor’s chambers were included in the private medical sector category.
	Pharmacy: Pharmacy/drug store was included in this category.
	Unqualified provider: Non-qualified doctor’s chamber was included in this category.
	NGO sector: The non-government organisation (NGO) sector category used to express the NGO sector health facilities as a source of advice and/or antibiotics were NGO statis clinic, NGO field worker, and another NGO sector.
	Other: The other category was used to express other health facilities including homeopaths and other traditional healers.

## Data Availability

The data presented in this study are available on request from the corresponding author.

## Supplementary Materials

The following are available online at https://www.mdpi.com/article/10.3390/antibiotics10101153/s1, Table S1: Multivariable logistic regression model to determine the association between age and antibiotic usage in children under five years old for any febrile illness, 2017-18 Bangladesh, Table S2: Multivariable logistic regression model to determine the association between wealth index and highest educational level of parent with antibiotic usage in children under five years old for any febrile illness, 2017-18 Bangladesh.
