# Supplementary material for: Antibiotic Use for Febrile Illness among Under-5 Children in Bangladesh: A Nationally Representative Sample Survey
Source: Antibiotics (Basel). 2021 Sep 24;10(10):1153. doi: 10.3390/antibiotics10101153 (PMC8532897; doi:10.3390/antibiotics10101153)
Supplement: Supplementary file 1 [file antibiotics-10-01153-s001.zip › antibiotics-1311146-supplementary.pdf]

Supplementary Table S1: Multivariable logistic regression model to determine the association between age and antibiotic usage in children under five years old for any febrile illness, 2017-18 Bangladesh

Model 2 –age without adjusting for other variables				
	Antibiotic usage in children under the age of five year with fever			
	UOR (95% CI)	p-value	AOR (95% CI)	p-value
**Age in months**				
<6	2.86 (1.83 - 4.47)	<0.001*		
44358	2.58 (1.62 - 4.11)	<0.001*		
44553	2.33 (1.49 - 3.65)	<0.001*		
24-35	1.49 (0.91 - 2.43)	0.11		
36-47	1.29 (0.79 - 2.11)	0.307		
48-59	Reference		Reference	
*A p-value less than 0.05 was significant				

Supplementary Table S2: Multivariable logistic regression model to determine the association between wealth index and highest educational level of parent with antibiotic usage in children under five years old for any febrile illness, 2017-18 Bangladesh

Model 3- wealth index adjusting for the effect of parents' highest education				
	Antibiotic usage in children under the age of five year with fever			
	UOR (95% CI)	p-value	AOR (95% CI)	p-value
**Wealth index**				
Poorest	0.90 (0.64 – 1.27)	0.552	1.15(0.8 - 1.64)	0.461
Poorer	0.87 (0.63 – 1.18)	0.374	1.01(0.72 - 1.42)	0.945
Middle	0.90 (0.66 – 1.24)	0.518	0.97(0.71 - 1.34)	0.870
Richest	0.72 (0.51 – 1.01)	0.058	0.66(0.46 - 0.93)	0.018*
Richer	Reference		Reference	
**Highest educational level of parent**				
No education	1.17 (0.56 - 2.44)	0.684	1.17 (0.56 - 2.44)	0.674
Primary complete	1.98 (1.22 - 3.22)	0.006*	2.04 (1.25 - 3.32)	0.004*
Secondary incomplete	1.80 (1.19 - 2.72)	0.006*	1.94 (1.26 - 2.98)	0.003*
Secondary complete or higher	2.15 (1.41 - 3.28)	<0.001*	2.61(1.63 - 4.16)	<0.001*
Primary incomplete	Reference		Reference	

*A p-value less than 0.05 was significant
